# Knowledge, attitude and practice toward oral anticoagulants among patients with atrial fibrillation

**DOI:** 10.3389/fcvm.2023.1301442

**Published:** 2023-12-15

**Authors:** Chang Li, Yubo Meng, Xiaoping Meng, Yuming Song

**Affiliations:** ^1^ Department of VIP Unit, China-Japan Union Hospital of Jilin University, Changchun, China; ^2^ Department of Research and Teaching, The Affiliated Hospital of Changchun University of Chinese Medicine, Changchun, China; ^3^ Department of Cardiac Rehabilitation, The Affiliated Hospital of Changchun University of Chinese Medicine, Changchun, China

**Keywords:** knowledge, attitude, practice, atrial fibrillation, oral anticoagulants, cross-sectional study

## Abstract

**Background:**

Atrial fibrillation (AF) is a common cardiac arrhythmia that increases the risk of stroke and other cardiovascular complications. Oral anticoagulants (OACs) are effective in reducing this risk. To investigate the knowledge, attitude and practice (KAP) toward OACs among patients with AF.

**Methods:**

This web-based cross-sectional study was conducted at local Hospital between April 2023 and May 2023, and enrolled AF patients.

**Results:**

A total of 491 valid questionnaires were collected, with 293 (59.67%) male and 73.93% resided in urban areas. The KAP scores were 4.64 ± 3.28, 21.09 ± 2.33 and 26.18 ± 2.15, respectively. Multivariate logistic regression analysis showed that junior high school [odd ratio (OR) = 0.346, 95% confidence interval (CI) = 0.145–0.825, *P* = 0.017], junior college/bachelor and above (OR = 6.545, 95% CI = 2.863–14.963, *P* < 0.001), monthly income ≥5,000 (OR = 2.343, 95% CI = 1.074–5.111, *P* = 0.032), never taken OACs (OR = 0.015, 95% CI = 0.004–0.059, *P* < 0.001), and having been diagnosed AF (6–10 months, OR = 4.003, 95% CI = 1.653–9.692, *P* = 0.002;over 20 months, OR = 4.046, 95% CI = 1.753–9.340, *P* = 0.001) were independently associated with knowledge. Knowledge (OR = 1.376, 95% CI = 1.162–1.629, *P* < 0.001), junior high school (OR = 0.258, 95% CI = 0.084–0.792, *P* = 0.018), monthly income ≥5,000 (OR = 5.486, 95% CI = 1.834–16.412, *P* = 0.002), and never undergone AF ablation (OR = 0.214, 95% CI = 0.097–0.471, *P* < 0.001) were independently associated with attitude. Knowledge (OR = 1.128, 95% CI = 1.030–1.235, *P* = 0.009), 70–79 years (OR = 2.193, 95% CI = 1.166–4.124, *P* = 0.015) and ≥80 years (OR = 4.375, 95% CI = 2.034–9.411, *P* < 0.001) were independently associated with proactive practice.

**Conclusion:**

Patients with AF had inadequate knowledge, suboptimal attitude and inactive practice towards AF and OACs. Improving patient education, especially among those with lower education levels, enhances understanding and management of AF and OACs.

## 1. Introduction

Atrial fibrillation (AF) is a cardiac arrhythmia characterized by irregular and rapid heartbeats, which is caused by abnormal electrical impulses in the atria of the heart (1, 2). It is a common condition that affects millions of people worldwide, mostly found in the older patients, and its prevalence is expected to increase due to the aging of the population (3). In China, it is estimated that ∼5.2 million men and ∼3.1 million women over the age of 60 will suffer from AF by the year 2050 (4). Therefore, proper management of AF is crucial for preventing complications, improving quality of life, and reducing the risk of stroke and other cardiovascular events.

One of the main treatments for AF is the use of oral anticoagulants (OACs) such as warfarin and non-vitamin K antagonist oral anticoagulants (NOACs), which can prevent the formation of blood clots in the heart and reduce the risk of stroke and systemic embolism (5). Many studies suggested that OACs in AF patients may also reduce the risk of cognitive decline and dementia (6). However, the optimal use of OACs requires proper patient education and engagement in the management of their condition (7). AF patients need to be aware of their condition, understand the risks and benefits of OACs, and adhere to the prescribed treatment regimen to achieve the best outcomes (8).

In the context of AF, evaluating patients' KAP related to their condition and anticoagulant therapy is essential for promoting optimal management and reducing the risk of complications, including stroke, bleeding and other cardiovascular events (9). It is important for developing effective patient education and engagement strategies to improve the management of AF and achieve better health outcomes. Therefore, this study aimed to investigate the KAP toward AF and OACs among patients with AF.

## 2. Methods

### 2.1. Study design and patients

The web-based cross-sectional study was conducted at China-Japan Union Hospital of Jilin University between April 2023 and May 2023, and AF patients were enrolled. Inclusion criteria were as following: (1) patients who diagnosed with non-valvular AF and (2) provided signed informed consent. The exclusion criteria were as follows: (1) patients with severe hemorrhage or other anticoagulant contraindications during outpatient or hospitalization. This study was approved by the Ethics Committee of China-Japan Union Hospital of Jilin University (No. 2023033013), and informed consent was obtained from patients before completing the questionnaire.

### 2.2. Procedures

A self-administered questionnaire was designed based on 2021 European Heart Rhythm Association Practical Guide on the Use of Non-Vitamin K Antagonist Oral Anticoagulants in Patients with Atrial Fibrillation (10), and reviewed by 3 cardiovascular disease experts. Similar or duplicate questions were removed, while others without clear explanation were adjusted and refined.

The final questionnaire included: (1) Demographic characteristics, including 10 items; (2) Knowledge dimension, including 10 items about AF and OACs, with 2 points for well known, 1 point for heard of, and 0 points for unclear; (3) Attitude dimension with 6 items, a five-point Likert scale was used, ranging from 5 points to 1 point from extremely positive to negative; (4) Practice dimension, including 15 questions, using the five-point Likert scale, ranging from always (5 points) to never (1 point). Higher scores are indicative of adequate knowledge, more positive attitude, and more proactive practice. A final score more than 75% of the total score indicates an adequate level of knowledge, a positive attitude, and proactive practice. A score ranging from 50% to 75% of the total score indicates a moderate level of knowledge, attitude, and practice. On the other hand, a score below 50% of the total score signifies inadequate knowledge, a negative attitude, and inactive practice (11).

A pre-test of 30 patients was conducted before the official distribution, with Cronbach's α of 0.916, indicating a high internal consistency. To ensure the reliability and validity of the study, rigorous measures were implemented to control sample quality and data accuracy. Exclusively outpatient or inpatient patients who met the inclusion criteria for atrial fibrillation were recruited, requiring confirmation of their condition by medical records or electrocardiogram (ECG) support. The study was conducted solely at the China-Japan Union Hospital of Jilin University to enhance consistency and comparability. Before initiating the study, two trained research assistants thoroughly explained the study's objectives and provided instructions to patients. During questionnaire distribution and retrieval, it was noted that some patients faced difficulties with the online questionnaire, leading us to provide a paper version and assigned research assistants to offer assistance. Wen Juan Xing (WJX) platform (https://www.wjx.cn) was used to create online questionnaires and generate quick response (QR) codes was used for data collection. Patients logged in and completed the questionnaire by scanning the provided QR code. To maintain data quality and completeness, only one submission per IP address was permitted, and all questions were mandatory. The research assistants provided clear explanations and instructions during the questionnaire completion process, ensuring patients' comprehension and accurate responses. They strictly adhered to principles of fairness, objectivity, and impartiality to avoid influencing patients' answers. The research team meticulously reviewed all questionnaires for completeness, internal consistency, and reasonableness.

### 2.3. Statistical analysis

Statistical analysis was performed using Stata 17.0 (Stata Corporation, College Station, TX, USA). Continuous data were expressed as mean ± standard deviation (SD) and compared by *t*-test or one-way ANOVA. The categorical data were presented as number and percentage [*n* (%)] and compared with the chi-square test. Pearson correlation was used to analyze the correlation of knowledge, attitude and practice scores. Variables with *P* < 0.05 in univariate analysis were included into multivariate analysis. Multivariate logistic regression analysis was performed to determine the factors associated with KAP, and 70% of the score distribution was used as cut-off value. Two-sided *P* < 0.05 was considered statistically significant.

## 3. Results

### 3.1. Demographic characteristics

A total of 501 questionnaires were initially collected for the study. However, after careful examination, 10 questionnaires were deemed unreasonable and excluded from the analysis. These exclusions were made based on specific criteria: one questionnaire had an abnormal age, six questionnaires reported a diagnosis of atrial fibrillation after the response time, and three questionnaires indicated the use of anticoagulant medication that did not correspond to the responses provided. As a result, the final dataset consisted of 491 valid questionnaires. Among them, 293 (59.67%) were male, 351 (71.49%) aged >60 years old, and 363 (73.93%) lived in urban areas. Approximately half of the patients (46.44%) had undergone AF ablation, and 291 (59.27%) were on OACs (Table 1).

**Table 1:** Baseline characteristics and KAP scores.

Variables	*N* (%)	Knowledge		Attitude		Practice	
		Mean ± SD	*P*	Mean ± SD	*P*	Mean ± SD	*P*
Total	491	4.64 ± 3.28		21.09 ± 2.33		26.18 ± 2.15	
Sex			0.044		0.112		<0.001
Male	293 (59.67)	4.88 ± 3.48		21.23 ± 2.45		25.90 ± 2.27	
Female	198 (40.33)	4.27 ± 2.94		20.89 ± 2.12		26.60 ± 1.90	
Age, years			0.042		0.230		<0.001
<60	140 (28.51)	4.94 ± 3.55		21.39 ± 2.42		25.69 ± 1.95	
60–69	163 (33.20)	4.98 ± 3.36		21.10 ± 2.41		25.97 ± 2.19	
70–79	120 (24.44)	4.25 ± 2.67		20.80 ± 2.18		26.37 ± 2.10	
≥80	68 (13.85)	3.88 ± 3.37		20.97 ± 2.13		27.37 ± 2.11	
Residence			<0.001		<0.01		0.427
Rural area	78 (15.89)	3.32 ± 2.05		20.28 ± 1.77		25.95 ± 2.07	
Urban area	363 (73.93)	5.08 ± 3.47		21.39 ± 2.34		26.26 ± 2.18	
Suburb area	50 (10.18)	3.44 ± 2.53		20.18 ± 2.46		26.00 ± 2.04	
Education			<0.001		<0.001		0.040
Primary school and below	66 (13.44)	2.76 ± 2.02		20.03 ± 1.70		26.62 ± 2.14	
Junior high school	120 (24.4)	2.99 ± 1.87		19.92 ± 1.86		26.07 ± 2.44	
High school/Technical secondary school	160 (32.59)	3.94 ± 2.38		20.91 ± 2.09		25.87 ± 1.99	
Junior college/Bachelor and above	145 (29.53)	7.63 ± 3.51		22.75 ± 2.23		26.42 ± 2.03	
Working status			<0.001		<0.001		0.004
Employed	67 (13.65)	6.66 ± 4.03		22.40 ± 5.62		26.15 ± 2.06	
Retired	196 (39.92)	5.19 ± 3.43		21.20 ± 2.37		26.55 ± 2.06	
Self-employed	32 (6.52)	5.06 ± 2.68		21.75 ± 2.33		25.19 ± 2.01	
Housewife	34 (6.92)	3.32 ± 2.11		20.76 ± 1.46		26.35 ± 1.74	
Other/Unemployed	162 (32.99)	3.32 ± 2.33		20.36 ± 1.99		25.91 ± 2.31	
Monthly income, CNY			<0.001		<0.001		0.321
<5,000	241 (49.08)	2.54 ± 1.87		19.66 ± 1.65		26.08 ± 2.26	
≥5,000	250 (50.92)	6.65 ± 3.08		22.47 ± 2.04		26.28 ± 2.04	
Marital status			0.558		0.683		0.143
Married	482 (98.17)	4.65 ± 3.28		21.10 ± 2.31		26.16 ± 2.14	
Widowed	9 (1.83)	4.00 ± 3.74		20.78 ± 3.27		27.22 ± 2.54	
Whether there are cohabitants			0.192		0.610		0.525
Yes	487 (99.19)	4.65 ± 3.28		21.10 ± 2.33		26.19 ± 2.15	
No	4 (0.81)	2.50 ± 3.70		20.50 ± 1.29		25.50 ± 1.91	
Medical insurance			<0.001		<0.001		0.015
Only social medical insurance	368 (74.95)	3.76 ± 2.87		20.52 ± 2.07		26.23 ± 2.20	
Both social medical insurance and commercial medical insurance	120 (24.44)	7.32 ± 3.07		22.92 ± 2.07		26.11 ± 1.92	
None of them	3 (0.61)	4.67 ± 2.52		18.00 ± 3.00		22.67 ± 2.89	
Undergone AF ablation			<0.001		<0.001		0.695
Yes	228 (46.44)	6.51 ± 3.01		22.35 ± 2.19		26.14 ± 1.91	
No	263 (53.56)	3.01 ± 2.57		20.00 ± 1.85		26.22 ± 2.34	
Taken OACs			<0.001		<0.001		0.647
Yes	291 (59.27)	6.48 ± 2.73		22.09 ± 2.18		26.14 ± 2.00	
No	200 (40.73)	1.95 ± 1.86		19.64 ± 1.68		26.24 ± 2.36	
Type of OACs	* *		<0.001		<0.001		0.403
Warfarin	4 (0.81)	10.50 ± 2.38		22.25 ± 0.50		27.50 ± 1.29	
NOAC	287 (58.45)	6.42 ± 2.70		22.08 ± 2.20		26.13 ± 2.00	
Duration of diagnosis of AF, months			<0.001		0.002		0.429
≤5	120 (24.44)	3.67 ± 3.22		20.93 ± 2.51		26.14 ± 1.94	
6–10	114 (23.22)	4.14 ± 2.47		20.46 ± 2.19		25.93 ± 2.41	
11–19	133 (27.09)	5.02 ± 3.41		21.50 ± 2.02		26.25 ± 2.07	
>20	124 (25.25)	5.61 ± 3.54		21.38 ± 2.44		26.38 ± 2.18	

AF, atrial fibrillation; OACs, oral anticoagulants; NOAC, new-oral-anticoagulants.

### 3.2. Knowledge, attitude and practice

The knowledge, attitude and practice scores were 4.64 ± 3.28 (possible range: 0–20), 21.09 ± 2.33 (possible range: 6–30) and 26.18 ± 2.15 (possible range: 7–35), respectively. Males (*P* = 0.044) and patients aged 60–70 years (*P* = 0.042) exhibited a higher likelihood of possessing better knowledge. On the other hand, females (*P* < 0.001) and older patients (*P* < 0.001) were more likely to demonstrate better practices. Moreover, the duration of AF diagnosis was found to influence knowledge and attitude. Patients who had been diagnosed with AF for a longer duration showed a greater likelihood of having better knowledge (*P* < 0.001) and a more positive attitude (*P* = 0.002). Regarding the choice of medication, patients who had taken warfarin displayed higher levels of knowledge and attitude compared to those who had taken NOACs (*P* < 0.001, respectively) (Table 1).

The distribution of knowledge indicated that patients did not achieve adequate knowledge regarding AF and OACs. Most patients, specifically 471 patients (95.93%), demonstrated a lack of clarity regarding the statement “During taking warfarin, the International Normalized Ratio (INR) should be monitored regularly and maintained between 2.0 and 3.0.” Interestingly, despite being the item with the highest number of respondents, only 76 patients (15.48%) selected “well known” in response to the statement “Patients with atrial fibrillation usually undergo anticoagulant therapy to prevent stroke and peripheral vascular embolism caused by thrombus shedding” (Table 2). Furthermore, it is worth noting that a significant number of patients were not aware of the impact of food and drugs on warfarin, as 87.78% of them chose the response “unclear” when presented with the statement “The effects of warfarin can be influenced by food and drugs. Stable diet should be ensured.”

**Table 2:** Knowledge, attitude and practice, *n* (%).

Statement			Well known	Heard	Unclear
1. Atrial fibrillation is the most common persistent arrhythmia, and its prevalence increases with the increase of age. Most patients are elderly, and stroke caused by atrial fibrillation is characterized by high prevalence, high disability rate and high mortality rate.			44 (8.96)	402 (81.87)	45 (9.16)
2. Patients with atrial fibrillation usually undergo anticoagulant therapy to prevent stroke and peripheral vascular embolism caused by thrombus shedding.			76 (15.48)	344 (70.06)	71 (14.46)
3. The CHA_2_DS_2_-VASc score is used to assess the risk of thromboembolism in patients with atrial fibrillation, and oral anticoagulation therapy should be used in men with ≥2 points or women with atrial fibrillation with ≥3 points			1 (0.20)	28 (5.70)	462 (94.09)
4. Oral anticoagulants commonly used include vitamin K antagonists (such as warfarin) and non-vitamin K antagonist oral anticoagulants (NOACs), but NOACs are preferred.			18 (3.67)	273 (55.60)	200 (40.73)
5. Warfarin is a classic anticoagulant, which is cheap and easy to buy.			6 (1.22)	97 (19.76)	388 (79.02)
6. During taking warfarin, the International Normalized ratio (INR) should be monitored regularly and maintained between 2.0 and 3.0			6 (1.22)	14 (2.85)	471 (95.93)
7. The effects of warfarin can be influenced by food and drugs. Stable diet should be ensured			6 (1.22)	54 (11.00)	431 (87.78)
8. Currently, NOAC listed in China include Dabigatran, Rivaroxaban and Edoxaban, while Apixaban has not been listed in China.			1 (0.20)	152 (30.96)	338 (68.84)
9. The anticoagulant effect of NOAC is stable and accurate, which is not affected by diet and has few interactions with other drugs. There is no need for frequent blood drawing to monitor INR or repeated adjustment of drug dosage.			57 (11.61)	225 (45.82)	209 (42.57)
10. The disadvantages of NOAC are increased the burden of patients with renal insufficiency and high price. Severe hemorrhage is troublesome if NOAC are taken in excess.			35 (7.13)	187 (38.09)	269 (54.79)
Attitude	Strongly agree	Agree	General	Disagree	Strongly disagree
1. I think anticoagulant therapy is a very important.	11 (2.24)	230 (46.84)	116 (23.63)	133 (27.09)	1 (0.20)
2. I have full trust in my primary care physician and it is very important to adjust the dosage of medication under their professional guidance.	10 (2.04)	279 (56.82)	170 (34.62)	31 (6.31)	1 (0.20)
3. I think anticoagulant treatment is very troublesome, especially warfarin. Thus, I don't want to take anticoagulant drugs.	56 (11.41)	187 (38.09)	124 (25.25)	123 (25.05)	1 (0.20)
4. NOAC is much more expensive than warfarin. Thus, I don't want to take NOAC	82 (16.70)	145 (29.53)	159 (32.38)	99 (20.16)	6 (1.22)
5. Whatever anticoagulant (warfarin or NOAC) I choose, I should pay close attention to hemorrhage tendencies.	17 (3.46)	468 (95.32)	6 (1.22)	0	0
6. I think non-active intervention might actually create more health care burden.	14 (2.85)	217 (44.20)	116 (23.63)	139 (28.31)	5 (1.02)
Practice	Strongly agree	Agree	Neutral	Disagree	Strongly disagree
1. The following factors may make you reject warfarin:					
1.1 Monitor relevant indicators regularly	46 (9.37)	433 (88.49)	11 (2.24)	1 (0.20)	0
1.2 Susceptible to other drugs, food, diseases, and medical conditions	283 (57.64)	205 (41.75)	3 (0.61)	0	0
1.3 Relatively high risk of intracranial hemorrhage	461 (93.89)	28 (5.70)	2 (0.41)	0	0
1.4 Need of giving up smoking and alcohol	6 (1.22)	102 (20.77)	99 (20.77)	113 (23.01)	171 (34.83)
2. The following factors will make you reject NOAC:					
2.1 Lack of specific antidotes	5 (1.02)	463 (94.30)	20 (4.07)	2 (0.41)	1 (0.20)
2.2 More likely for gastrointestinal bleeding and indigestion	150 (30.55)	334 (68.02)	5 (1.02)	2 (0.41)	0
2.3 High requirements for compliance	5 (1.02)	191 (38.90)	127 (25.87)	161 (32.79)	7 (1.43)
2.4 High price	80 (16.29)	181 (36.86)	142 (28.92)	85 (17.31)	3 (0.61)
	Always	Often	Sometimes	Rarely	Never
3. The frequency with which you can perform the following behaviors is:					
3.1 Maintain good medication compliance to avoid missing drug use	8 (1.63)	173 (35.23)	263 (53.56)	47 (9.57)	0
3.2 Active follow-up review	1 (0.20)	80 (16.29)	210 (42.77)	198 (40.33)	2 (0.41)
3.3 Proactively obtain information about atrial fibrillation from doctors	0	52 (10.59)	149 (30.35)	257 (52.34)	33 (6.72)
3.4 Get enough rest and sleep	2 (0.41)	302 (60.51)	186 (37.88)	1 (0.20)	0
3.5 Exercise moderately and avoid strenuous exercise	1 (0.20)	112 (22.81)	241 (49.08)	135 (27.49)	2 (0.41)
3.6 Eat healthy and avoid irritating foods	1 (0.20)	318 (64.77)	169 (34.42)	3 (0.61)	0
3.7 Use over-the-counter medications with caution	21 (4.28)	225 (45.82)	242 (49.29)	3 (0.61)	0

The attitude distribution revealed that most patients exhibited a generally positive attitude towards AF and OACs. However, the proportion of patients who chose “Strongly Agree” was relatively low. For example, only 16.7% strongly agreed with the statement “NOAC is much more expensive than warfarin. Thus, I don't want to take NOAC,” and 2.04% strongly agreed with the statement “I have full trust in my primary care physician, and it is very important to adjust the dosage of medication under their professional guidance” (Table 2).

In the practice dimension, most patients (93.83%) chose the reason “Relatively high risk of intracranial hemorrhage” as the primary factor for rejecting warfarin. On the other hand, for rejecting NOACs, the main factor selected was “More likely for gastrointestinal bleeding and indigestion,” with 30.55% considering it as “Strongly agree” and 68.02% rating it as “Agree” (Table 2).

### 3.3. Pearson correlation analysis

Pearson correlation analysis showed that knowledge was positively associated with attitude (*r* = 0.648, *P* < 0.001) and practice (*r* = 0.113, *P* = 0.012). There was no significant correlation between attitude and practice (*r* = 0.077, *P* = 0.088) (Table 3).

**Table 3:** Pearson correlation analysis.

	Knowledge	Attitude	Practice
Knowledge	1		
Attitude	0.648 (*P* < 0.001)	1	
Practice	0.113 (*P* = 0.012)	0.077 (*P* = 0.088)	1

### 3.4. Multivariate logistic regression

The results of the multivariate logistic regression analysis indicated several independent associations with knowledge, attitude and practice. Junior high school education level (odds ratio [OR] = 0.346, 95% confidence interval [CI] = 0.145–0.825, *P* = 0.017), and never having taken oral anticoagulants (OR = 0.015, 95% CI = 0.004–0.059, *P* < 0.001) were independently associated with poor knowledge. Whereas junior college/bachelor and above education level (OR = 6.545, 95% CI = 2.863–14.963, *P* < 0.001), a monthly income of over 5,000 (OR = 2.343, 95% CI = 1.074–5.111, *P* = 0.032), a longer diagnosis duration of AF (6–10 months, OR = 4.003, 95% CI = 1.653–9.692, *P* = 0.002; over 20 months, OR = 4.046, 95% CI = 1.753–9.340, *P* = 0.001) were found to be independently associated with adequate knowledge. Knowledge (OR = 1.376, 95% CI = 1.162–1.629, *P* < 0.001), a monthly income of ≥5,000 (OR = 5.486, 95% CI = 1.834–16.412, *P* = 0.002), and no previous AF ablation (OR = 0.214, 95% CI = 0.097–0.471, *P* < 0.001) were independently associated with positive attitude. However, junior high school education level (OR = 0.258, 95% CI = 0.084–0.792, *P* = 0.018) was independently associated with negative attitude. Knowledge (OR = 1.128, 95% CI = 1.030–1.235, *P* = 0.009), age group of 70–79 years (OR = 2.193, 95% CI = 1.166–4.124, *P* = 0.015), and age group of ≥80 years (OR = 4.375, 95% CI = 2.034–9.411, *P* < 0.001) were independently associated with proactive practice (Table 4).

**Table 4:** Univariate and multivariate logistic regression analysis.

	Variables	Univariate analysis		Multivariate analysis	
		OR (95% CI)	*P*	OR (95% CI)	*P*
Knowledge	Gender				
	Male	Ref.		Ref.	
	Female	0.572 (0.367–0.890)	0.013	1.118 (0.571–2.189)	0.657
	Age, years				
	>60	Ref.			
	60–69	1.128 (0.680–1.871)	0.641		
	70–79	0.625 (0.344–1.134)	0.122		
	≥80	0.597 (0.288–1.235)	0.164		
	Residence				
	Rural area	0.202 (0.085–0.479)	<0.001	0.514 (0.190–1.389)	0.460
	Urban area	Ref.		Ref.	
	Suburb area	0.331 (0.137–0.799)	0.014	0.690 (0.256–1.861)	0.661
	Education				
	Primary school and below	0.344 (0.146–0.814)	0.015	0.634 (0.209–1.921)	0.420
	Junior high school	0.264 (0.126–0.552)	<0.001	0.346 (0.145–0.825)	0.017
	High school/Technical secondary school	Ref.		Ref.	
	Junior college/Bachelor and above	8.472 (5.061–14.181)	<0.001	6.545 (2.863–14.963)	<0.001
	Working status				
	Employed	Ref.		Ref.	
	Retired	0.477 (0.271–0.839)	0.010	1.187 (0.429–3.286)	0.741
	Self-employed	0.238 (0.087–0.652)	0.005	0.788 (0.209–2.969)	0.725
	Housewife	0.064 (0.014–0.291)	<0.001	0.763 (0.152–3.817)	0.741
	Other/Unemployed	0.105 (0.051–0.215)	<0.001	0.854 (0.261–2.801)	0.795
	Monthly income, CNY				
	<5,000	Ref.		Ref.	
	≥5,000	16.676 (9.797–28.384)	<0.001	2.343 (1.074–5.111)	0.032
	Marital status				
	Married	Ref.			
	Widowed	1.596 (0.393–6.481)	0.513		
	Undergone AF ablation				
	Yes	Ref.		Ref.	
	No	0.162 (0.100–0.263)	<0.001	0.958 (0.469–1.957)	0.958
	Taken OACs				
	Yes	Ref.		Ref.	
	No	0.023 (0.007–0.075)	<0.001	0.015 (0.004–0.059)	<0.001
	Duration of diagnosis of AF, months				
	≤5	Ref.		Ref.	
	6–10	0.760 (0.392–1.472)	0.416	4.003 (1.653–9.692)	0.002
	11–20	1.155 (0.636–2.097)	0.636	1.343 (0.600–3.004)	0.473
	>20	2.017 (1.135–3.586)	0.017	4.046 (1.753–9.340)	0.001
Attitude	Knowledge	1.916 (1.693–2.168)	<0.001	1.376 (1.162–1.629)	<0.001
	Gender				
	Male	Ref.		Ref.	
	Female	0.458 (0.299–0.703)	<0.001	0.573 (0.277–1.182)	0.132
	Age, years				
	>60	Ref.		Ref.	
	60–69	0.775 (0.478–1.257)	0.302	1.509 (0.545–4.176)	0.428
	70–79	0.489 (0.279–0.856)	0.012	1.862 (0.545–6.357)	0.321
	≥80	0.571 (0.296–1.105)	0.096	4.097 (0.926–18.133)	0.063
	Residence				
	Rural area	0.163 (0.069–0.385)	<0.001	0.433 (0.132–1.415)	0.270
	Urban area	Ref.		Ref.	
	Suburb area	0.428 (0.202–0.910)	0.027	0.891 (0.280–2.828)	0.786
	Education				
	Primary school and below	0.170 (0.050–0.575)	0.004	0.205 (0.041–1.015)	0.052
	Junior high school	0.188 (0.076–0.463)	<0.001	0.258 (0.084–0.792)	0.018
	High school/Technical secondary school	Ref.		Ref.	
	Junior college/Bachelor and above	6.583 (3.966–10.925)	<0.001	1.580 (0.635–3.932)	0.465
	Working status				
	Employed	Ref.		Ref.	
	Retired	0.361 (0.205–0.638)	<0.001	0.403 (0.114–1.425)	0.159
	Self-employed	0.673 (0.289–1.569)	0.360	1.267 (0.311–5.161)	0.742
	Housewife	0.048 (0.011–0.216)	<0.001	0.276 (0.037–2.036)	0.207
	Other/Unemployed	0.107 (0.055–0.211)	<0.001	0.553 (0.150–2.043)	0.374
	Monthly income, yuan				
	<5,000	Ref.		Ref.	
	≥5,000	53.655 (21.384–134.627)	<0.001	5.486 (1.834–16.412)	0.002
	Marital status				
	Married	Ref.			
	Widowed	0.727 (0.149–3.543)	0.693		
	Undergone AF ablation				
	Yes	Ref.		Ref.	
	No	0.077 (0.045–0.130)	<0.001	0.214 (0.097–0.471)	<0.001
	Taken OACs				
	Yes	Ref.		Ref.	
	No	0.037 (0.016–0.087)	<0.001	0.920 (0.243–3.480)	0.625
	Duration of diagnosis of AF, months				
	≤5	Ref.			
	6–10	0.595 (0.320–1.107)	0.101		
	11–20	1.303 (0.760–2.235)	0.336		
	>20	1.255 (0.724–2.176)	0.417		
Practice	Knowledge	1.070 (1.009–1.135)	0.024	1.128 (1.030–1.235)	0.009
	Attitude	1.010 (0.928–1.099)	0.821	0.900 (0.799–1.013)	0.082
	Gender				
	Male	Ref.			
	Female	1.446 (0.973–2.151)	0.068		
	Age, years				
	>60	Ref.		Ref.	
	60–69	1.678 (0.956–2.944)	0.071	1.515 (0.845–2.718)	0.163
	70–79	2.327 (1.300–4.166)	0.004	2.193 (1.166–4.124)	0.015
	≥80	4.833 (2.530–9.235)	<0.001	4.375 (2.034–9.411)	<0.001
	Residence				
	Rural area	0.448 (0.237–0.846)		0.492 (0.238–1.019)	0.056
	Urban area	Ref.	0.013	Ref.	
	Suburb area	0.872 (0.452–1.680)	0.681	0.981 (0.484–2.222)	0.957
	Education				
	Primary school and below	2.705 (1.440–5.081)	0.002	1.901 (0.881–4.104)	0.102
	Junior high school	1.645 (0.942–2.874)	0.080	1.557 (0.846–2.863)	0.155
	High school/Technical secondary school	Ref.		Ref.	
	Junior college/Bachelor and above	2.059 (1.221–3.473)	0.007	1.627 (0.875–3.025)	0.124
	Working status				
	Employed	Ref.			
	Retired	1.342 (0.731–2.463)	0.342		
	Self-employed	0.468 (0.157–1.394)	0.173		
	Housewife	0.909 (0.359–2.302)	0.841		
	Other/Unemployed	0.774 (0.407–1.471)	0.436		
	Monthly income, yuan				
	<5,000	Ref.			
	≥5,000	1.050 (0.709–1.556)	0.806		
	Marital status				
	Married	Ref.			
	Widowed	2.056 (0.544–7.773)	0.288		
	Undergone AF ablation				
	Yes	Ref.			
	No	1.476 (0.990–2.200)	0.056		
	Taken OACs				
	Yes	Ref.			
	No	1.249 (0.839–1.857)	0.273		
	Duration of diagnosis of AF, months				
	≤5	Ref.			
	6–10	1.071 (0.596–1.927)	0.818		
	11–20	1.385 (0.797–2.404)	0.248		
	>20	1.276 (0.725–2.404)	0.398		

AF, atrial fibrillation; OACs, oral anticoagulants; NOAC, new-oral-anticoagulants.

## 4. Discussion

Patients with AF showed inadequate knowledge, suboptimal attitude and inactive practice towards AF and OACs. Education level, income, knowledge, and age were found to be factors associated with the KAP of patients in managing AF. Specially, the patients with higher education level, higher household income, a longer duration are associated with more adequate knowledge. Notably, the knowledge of AF is positively associated the practice. Overall, these findings highlight the need for improved education and awareness among patients with AF regarding their condition and treatment options.

The study's findings highlighted a significant knowledge and practice gap in the management of AF among patients, which is consistent with the results of previous studies conducted by the European Heart Rhythm Association (12). It is concerning that not only patients but also some healthcare professionals lack adequate knowledge in this area. For instance, a KAP study among primary care physicians in China found that 75.8% of the patients had insufficient knowledge of OACs therapy for patients with non-valvular AF (13). Particularly, neurologists were also found to have significant gaps in their understanding and practice regarding AF management (14). One notable finding was the apparent lack of awareness among patients with AF. Specially, more than 94% of the AF patients demonstrated a lack of clarity regarding the CHA2DS2-VASc score and International Normalized Ratio (INR) in this study. Inaccurate understanding of the CHA2DS2-VASc score might lead patients to underestimate their risk of stroke, which could result in inadequate preventive measures and an elevated likelihood of stroke occurrence. Furthermore, misinterpretation of the INR could lead to inappropriate adjustments in anticoagulant dosages, putting patients at increased risk of bleeding or clotting events. It emphasizes the need for healthcare providers to prioritize patient education and provide clear explanations about the potential unfavorable consequences, ensuring that AF patients are well-informed about their condition and the associated risk assessment tools and treatment options.

The results of this study highlight the importance of education level in the management of AF. Our findings showed that patients with higher education levels had better knowledge and practice of AF and OACs. Knowledge scores were relatively low, particularly among patients with lower education levels and those who had not received OACs. This is consistent with previous studies, where education level was found to be the strongest predictor of reporting high disease understanding of AF among medical history and demographic factors (15). Similarly, a study also showed that education level was an important factor associated with the knowledge of AF and OACs (14). A higher education level often correlates with better health literacy, allowing patients to comprehend complex medical information, treatment options, and the importance of oral anticoagulants OACs. Previous study showed that the lack of sufficient education and training on AF and OACs among lower-educated patients resulted in poor knowledge and practice (16). Education programs that target both patients and healthcare providers can improve the quality of care and management of AF (17). Therefore, providing easy-to-understand information and resources on AF and OACs is important to improve patient knowledge and practice especially for those with lower education level.

Furthermore, the study identified an area of concern regarding patient attitudes towards AF and OACs. While overall attitudes were moderately favorable, patients with lower education levels and those who had never undergone AF ablation exhibited suboptimal attitudes. Addressing misconceptions and concerns through patient consultations and educational interventions can play a pivotal role in shaping patients' attitudes and their acceptance of necessary medical procedures (18, 19). On a positive note, the study revealed encouraging results regarding proactive practices among the surveyed patients. Proactive practice scores were relatively high, especially among older age groups. These findings suggest that older patients might have a more responsible approach towards their health management (20). Emphasizing the importance of proactive self-management and adherence to OACs, regardless of age, could benefit patients across all age groups and help in reducing the risk of stroke and other cardiovascular complications associated with AF.

The observation that longer durations of AF diagnosis, specifically extending beyond 20 months were independently associated with adequate knowledge. This finding may reflect a potential learning curve that patients experience over time, as they navigate the complexities of their condition (21). As patients accumulate more experience with AF, they likely encounter various sources of information, engage more frequently with healthcare professionals, and develop a deeper understanding of the condition's implications and management requirements. Longer diagnosis durations might provide ample opportunities for patients to seek education, ask questions, and gain insights from their interactions with medical experts. Consequently, this enhanced knowledge could empower patients to better grasp crucial aspects of AF, such as its potential complications, treatment options, and the role of oral anticoagulants. These findings underline the importance of early and continuous education and support initiatives for patients newly diagnosed with AF, with the goal of accelerating their understanding and decision-making, while also emphasizing the need for ongoing education for all AF patients to maintain their knowledge and engagement over time.

Current guidelines strongly recommend OACs therapy for AF patients with one or more risk factors for stroke, such as older age, prior stroke or transient ischemic attack, hypertension, heart failure, diabetes, and vascular disease (22, 23). However, this study revealed a concerning finding that only 59.27% of patients had ever taken OACs, which is lower than expected based on current guidelines. This is consistent with a study in Poland involving elderly AF patients at admission, where only 58.9% received oral anticoagulants at the time of admission and the severe frailty and the presence of anemia reduced the percentage of use of OACs (24). In this study, the main reason reported for rejecting warfarin was the relatively high risk of intracranial hemorrhage. However, the rejection of NOACs was attributed to several concerns, including the absence of specific antidotes and an increased risk of gastrointestinal bleeding and indigestion. Warfarin, a classic anticoagulant with a long history of use, presents a key challenge due to the requirement for frequent INR monitoring to maintain stability. It's noteworthy that a significant majority of patients (95.93%) demonstrated a lack of knowledge regarding INR monitoring on warfarin treatment. Additionally, it's important to acknowledge that understanding renal insufficiency and the necessity for dosage adjustments is crucial not only for warfarin but also for other oral anticoagulants (OACs) and warrants further investigation.

A multifaceted and multilevel educational intervention did result in a significant increase in the proportion of AF patients treated with OACs (25). Therefore, it is crucial for healthcare providers to address these concerns and provide accurate information about the available management strategies for OACs, as well as the overall safety profile and effectiveness of OACs in stroke prevention (26).

Enhancing patients' understanding and adherence regarding AF and OACs remains a pivotal facet of comprehensive management. These imperative gains even more significance on strategies that prioritize patient-centric education, encompassing illuminative workshops and easily accessible online resources designed to demystify AF, expound upon the significance of OACs, and elucidate the relevance of the CHA2DS2-VASc score (27). Moreover, the introduction of mobile and internet management introduces promising avenues for remote patient monitoring, tailored information dissemination, and facilitating timely communication, thereby amplifying patient engagement and adherence to treatment regimens. In conclusion, the pursuit of heightening patient comprehension and adherence in the landscape of AF and OAC management mandates an encompassing and multifaceted approach, wherein education, communication, support, counseling, monitoring, and technological innovations synergistically coalesce to empower patients and ultimately enhance their clinical outcomes.

This study has certain limitations that need to be acknowledged. Firstly, the study was conducted in a single center and with a relatively small sample size, which may limit the generalizability of the results to other regions. Thus, further studies with larger and more diverse samples are necessary to validate these findings. Additionally, as this was a cross-sectional study, it only provides KAP at a specific time point and cannot establish causality. Longitudinal studies would be beneficial in assessing the changes in KAP over time and their effect on clinical outcomes. Furthermore, the patient's oral anticoagulant time and compliance is also important to know their knowledge and practice of AF and OACs. However, they were not included in the questionnaire for the time constraints during the cross-sectional survey and considerations related to patient patience and compliance.

In conclusion, patients with AF had inadequate knowledge, moderate attitude and inactive practice towards AF and OACs. Improving patient education, especially among those with lower education levels, enhances understanding and management of AF and OACs.

## Data Availability

The original contributions presented in the study are included in the article/Supplementary Material, further inquiries can be directed to the corresponding authors.
